# Residues of acidic chitinase cause chitinolytic activity degrading chitosan in porcine pepsin preparations

**DOI:** 10.1038/s41598-019-52136-2

**Published:** 2019-10-30

**Authors:** Eri Tabata, Satoshi Wakita, Akinori Kashimura, Yasusato Sugahara, Vaclav Matoska, Peter O. Bauer, Fumitaka Oyama

**Affiliations:** 1 Department of Chemistry and Life Science, Kogakuin University, Hachioji, Tokyo 192-0015 Japan; 2 Research Fellow of Japan Society for the Promotion of Science (DC1), Koujimachi, Chiyoda-ku, Tokyo 102-0083 Japan; 3 Laboratory of Molecular Diagnostics, Department of Clinical Biochemistry, Hematology and Immunology, Homolka Hospital, Roentgenova 37/2, Prague, 150 00 Czech Republic; 4 Bioinova Ltd., Videnska 1083, Prague, 142 20 Czech Republic

**Keywords:** Dietary carbohydrates, Polysaccharides, Hydrolases, Dietary carbohydrates

## Abstract

Commercially available porcine pepsin preparations have been used for the production of chitooligosaccharides with various biomedical activities. However, the origin of this activity is not well understood. Here we show that the chitosan-degrading activity is conferred by residues with chitinolytic activity of truncated forms of acidic chitinase (Chia) persisting in the pepsin preparation. Chia is an acid-stable and pepsin-resistant enzyme that degrades chitin to produce *N*-acetyl-D-glucosamine dimer. We found that Chia can be truncated by pepsin under stomach-like conditions while maintaining its enzymatic activity. Similarly to the full-length protein, truncated Chia as well as the pepsin preparations digested chitosan with different degrees of deacetylation (DD: 69–84%) with comparable degradation products. The efficiency was DD-dependent with a marked decrease with higher DD, indicating that the chitosan-degrading activity in the pepsin preparation is due to the chitinolytic activity rather than chitosanolytic activity. We suggest that natural or recombinant porcine Chia are suitable for producing chitooligosaccharides for biomedical purposes.

## Introduction

Chitin is a polymer of β-1, 4-linked *N*-acetyl-D-glucosamine (GlcNAc), which is an integral component of the exoskeleton of crustaceans and insects, the microfilarial sheaths of parasites and the cell walls in fungi^1–3^. Chitosan, a partially deacetylated derivative of chitin, is a heteropolymer of D-glucosamine (GlcN) and GlcNAc residues. In nature, this polymer is partially acetylated, and the term “chitosan” describes a large family of polymers with various GlcN/GlcNAc ratios.

Acidic chitinase (hereafter referred to as “Chia”; also reported as acidic mammalian chitinase, “AMCase”) is a 50 kDa enzyme that is expressed primarily in the stomach tissues and degrades β-1, 4 bonds of chitin^4–8^. Recently, we showed that Chia functions as a protease-resistant glycosidase under gastrointestinal conditions in mouse, chicken, porcine and common marmoset^9–12^. Chia can be purified by chitin chromatography by elution with urea or acetic acid^11,13^ and the isolated enzyme produces (GlcNAc)_2_ fragments in the porcine gastrointestinal environment^13^.

Chitooligosaccharides are homo- or heterooligomers containing GlcN and GlcNAc residues. They attracted substantial interest due to their diverse biomedical activities such as anti-microbial^14^, hypocholesterolemic^15^, anti-inflammatory^16^, anti-tumor effects^17^, drug delivery^18^ and accelerating calcium and iron absorption^19^.

Chitooligosaccharides have been produced by several methods such as acid hydrolysis and enzymatic degradation^20,21^. Enzymatic preparation methods using mammalian sources have a high potential of enhancing the value of the products due to their safety and simplicity of the process control^22^. Many nonspecific enzymes, such as cellulases, lipases and proteases as well as chitosanases, have been used to prepare chitooligosaccharides^22–24^. Although commercially available porcine pepsin preparations have been shown to possess chitosanolytic activity^22,25–27^, the origin of such activity has not been well understood.

We found that this activity results from the presence of fragments of Chia truncated by pepsin under stomach conditions and retaining the chitin/chitosan-degrading activity. Furthermore, full-length and truncated Chia, as well as pepsin preparations, have similar efficacy in terms of the composition of the resulting chitooligosaccharides.

## Results

### Degradation of chitosan by pepsin preparations

Commercially available porcine pepsin preparations have been shown to degrade chitosan into chitooligosaccharides^22,25–27^, suggesting that pepsin may have chitosanolytic (chitosan-degrading) activity. This being a contra-intuitive hypothesis, we first explored the entity of this activity by exposing chitosan (degree of deacetylation, DD 80%) as well as α- or β-crystalline chitin (DD 2% or 10%, respectively) to a pepsin preparation (Sigma-Aldrich, P7012) or purified pepsin A (Pep A, Worthington) at pH 2.0 or pH 4.0.

**Figure 1:**
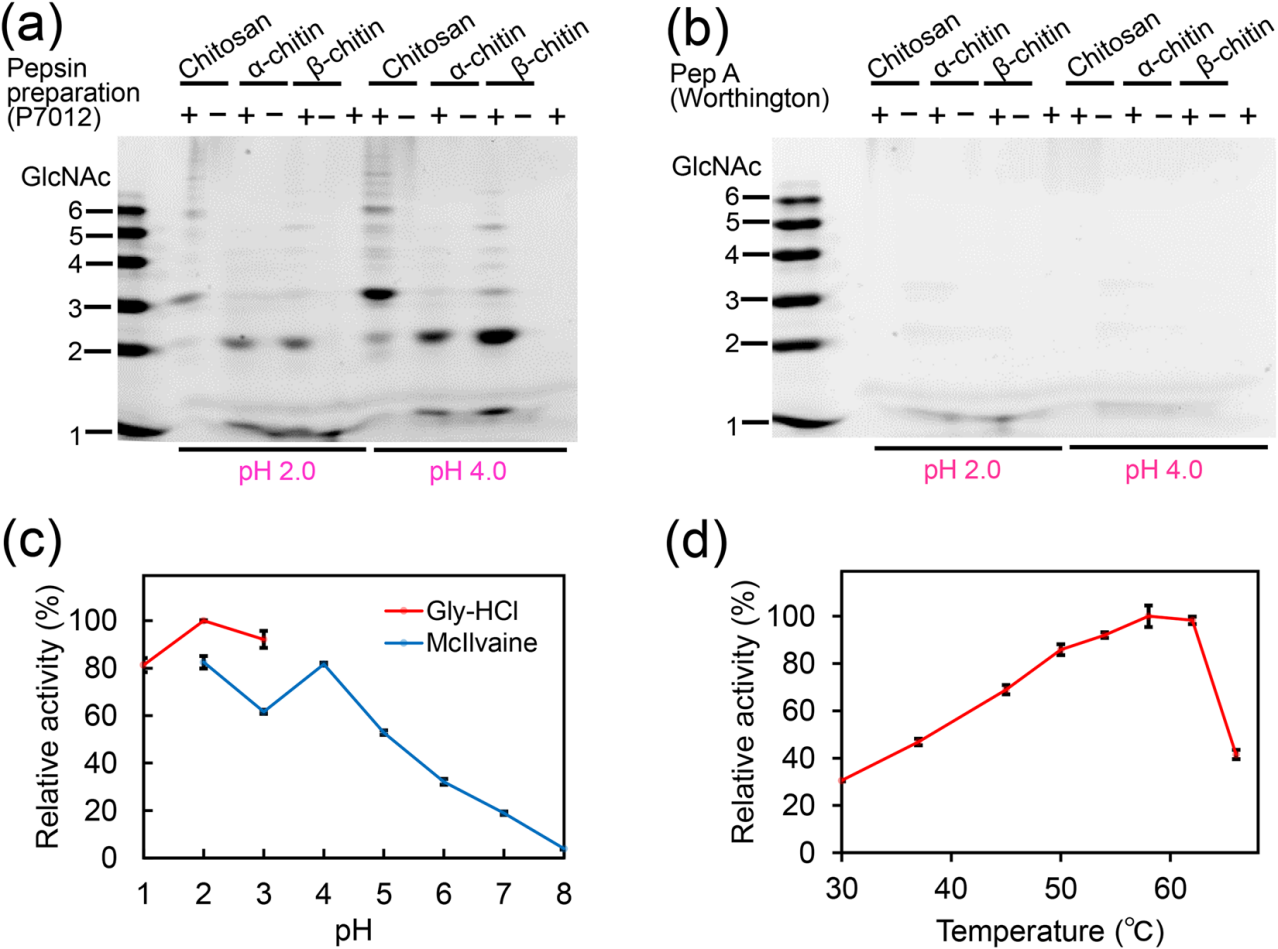
Pepsin preparation P7012 possesses chitinolytic activity as well as chitosan-degrading activity. Degradation products generated by incubation of 80% DD chitosan, α- or β-chitin with (**a**) pepsin preparation (P7012) or (**b**) Pep A at pH 2.0 or pH 4.0 were analyzed by FACE. The images of (**a**,**b**) were cropped from dotted lines on original full-length gel images shown in Supplementary Fig. S1. (**c**) Optimal pH or (**d**) optimal temperature of the chitinolytic activity in the pepsin preparation was measured with 4-NP-(GlcNAc)_2_. Values in (**c**,**d**) represent mean ± SD conducted in triplicate.

The resulting oligosaccharides were analyzed by fluorophore-assisted carbohydrate electrophoresis (FACE) as described in Methods. The pepsin preparation degraded chitosan producing oligomers with mobility similar to (GlcNAc)_3_ as well as species longer than (GlcNAc)_6_ (Fig. 1a and Supplementary Fig. S1). Unexpectedly, it also degraded α-chitin to produce (GlcNAc)_2_ (Fig. 1a). As for β-chitin, the pepsin preparation produced (GlcNAc)_2_ and (GlcNAc)_3__–__6_ (Fig. 1a). These substrates were more efficiently degraded by the preparation at pH 4.0 as compared to pH 2.0 (Fig. 1a).

On the other hand, Pep A did not affect any of the substrates (Fig. 1b and Supplementary Fig. S1). These results indicate that the pepsin preparations may possess chitosan-degrading as well as chitinolytic activity originating from molecules other than pepsin.

We further characterized the observed chitinolytic activity in the pepsin preparation using 4-nitrophenyl *N*,*N’*-diacetyl-β-D-chitobioside [4-NP-(GlcNAc)_2_], a synthetic substrate widely used for measuring such activity. The reactions were performed at different pH in 0.1 M Gly-HCl (pH 1.0–3.0) or McIlvaine’s (pH 2.0–8.0) buffers. The highest activity was detected at pH 2.0 in 0.1 M Gly-HCl buffer. In McIlvaine’s buffer, high enzymatic activity was observed at pH 2.0–5.0 with peaks at pH 2.0 and pH 4.0 with a gradual decrease in less acidic environments (pH 6.0–8.0) (Fig. 1c). The optimal pH at 2.0–4.0 has previously been described as a characteristic feature of the chitinolytic activity of the porcine Chia^11^.

The effect of temperature on the enzymatic activity was determined in 0.1 M Gly-HCl buffer at pH 2.0 and temperatures ranging from 30–64 °C using the same substrate for 30 min. The rate of the pepsin preparation-catalyzed reaction was gradually enhanced with increasing temperature and reached the maximum level at 58 °C, then abruptly decreased (Fig. 1d), copying the pattern seen in porcine Chia^11^.

### Detection of protease-resistant truncated porcine Chia in the pepsin preparation

**Figure 2:**
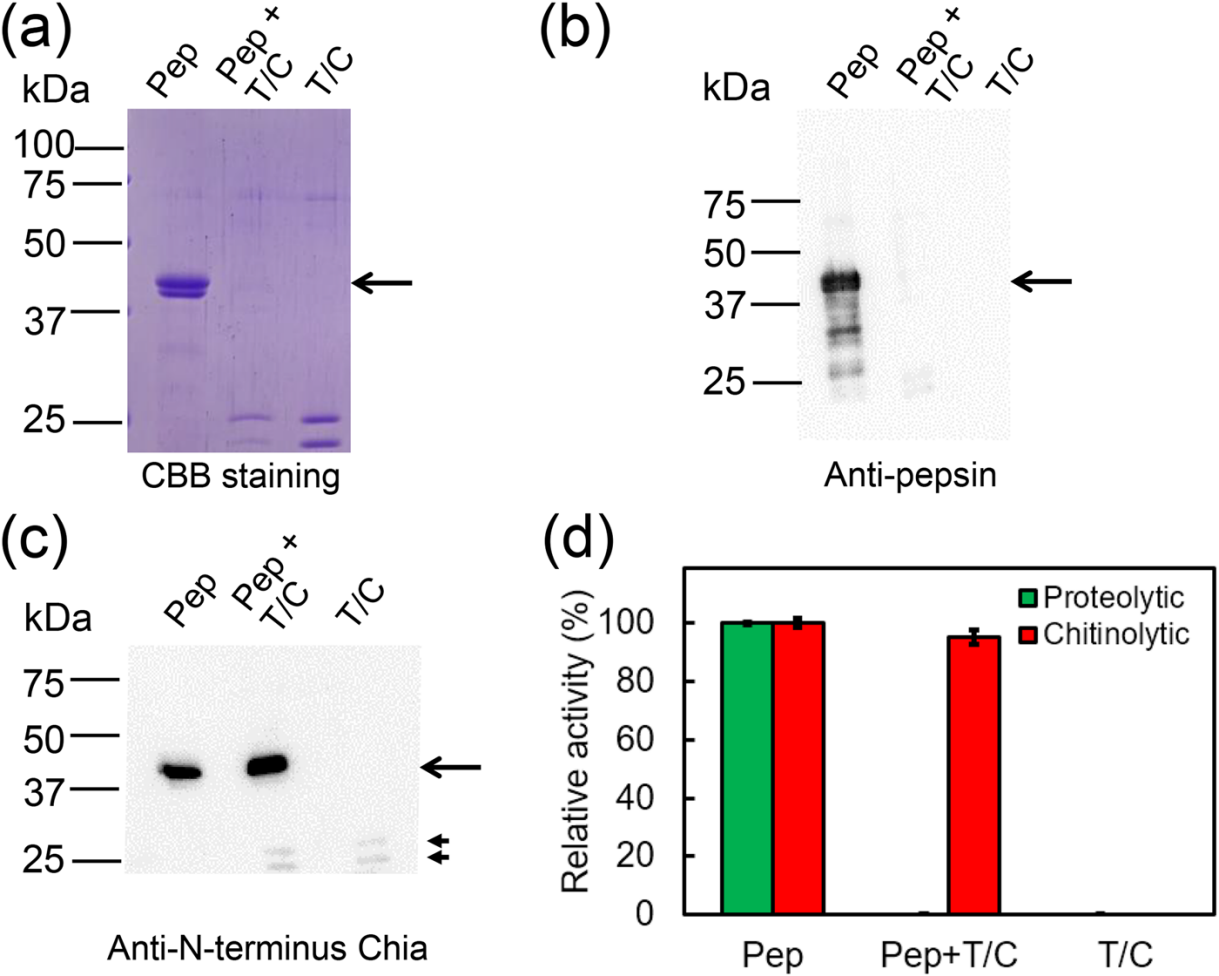
Detection of the truncated porcine Chia and its chitinolytic activity. Porcine pepsin preparation [P7012 (Pep)] was incubated with trypsin and chymotrypsin (T/C) at 37 °C for 10 min at pH 7.6. (**a**) Total protein analysis by CBB staining, WB using (**b**) anti-porcine pepsin antibody or (**c**) anti-porcine N-terminus Chia antibody. The specific bands (shown by arrowheads) may result from cross-reaction of trypsin/chymotrypsin with the antibody. The images of (**a**–**c**) were cropped from original full-length gel images shown in dotted lines in Supplementary Fig. S2. (**d**) Chitinolytic activities in the pepsin preparation without or with trypsin and chymotrypsin treatment were measured at pH 2.0 as described in Methods. Values in (**d**) represent mean ± SD from a single experiment conducted in triplicate.

Previously, high expression of Chia mRNA in the porcine stomach, the enzyme’s resistance to proteases and the highest activity at pH 2.0–4.0 and 58 °C have been reported^11^. We hypothesized that the chitosan-degrading activity in the pepsin preparations can be attributed to residual Chia. To verify this assumption, the P7012 pepsin preparation was incubated with trypsin and chymotrypsin at pH 7.6 for 10 min and analyzed by SDS-PAGE, followed by CBB staining or Western blot (WB). As expected, pepsin was degraded after this treatment (Fig. 2a and b; Supplementary Fig. S2). The Chia, however, remained present after the treatment (Fig. 2c and Supplementary Fig. S2). The pepsin preparation was subjected to WB using antibody against the N-terminus of Chia and the presence of the enzyme was confirmed (Fig. 2c, first lane; Supplementary Fig. S2).

To analyze the functional effect of trypsin/chymotrypsin treatment, we measured proteolytic and chitinolytic activities in the pepsin preparation at pH 2.0. Both activities were detected in the intact pepsin preparation, but only chitinolytic activity remained present after the treatment (Fig. 2d). These results suggest that chitosan-degrading activity in the pepsin preparation is due to the chitinolytic activity of the truncated porcine Chia.

### The degradation efficiency of chitosan varies depending on the porcine pepsin preparations

**Figure 3:**
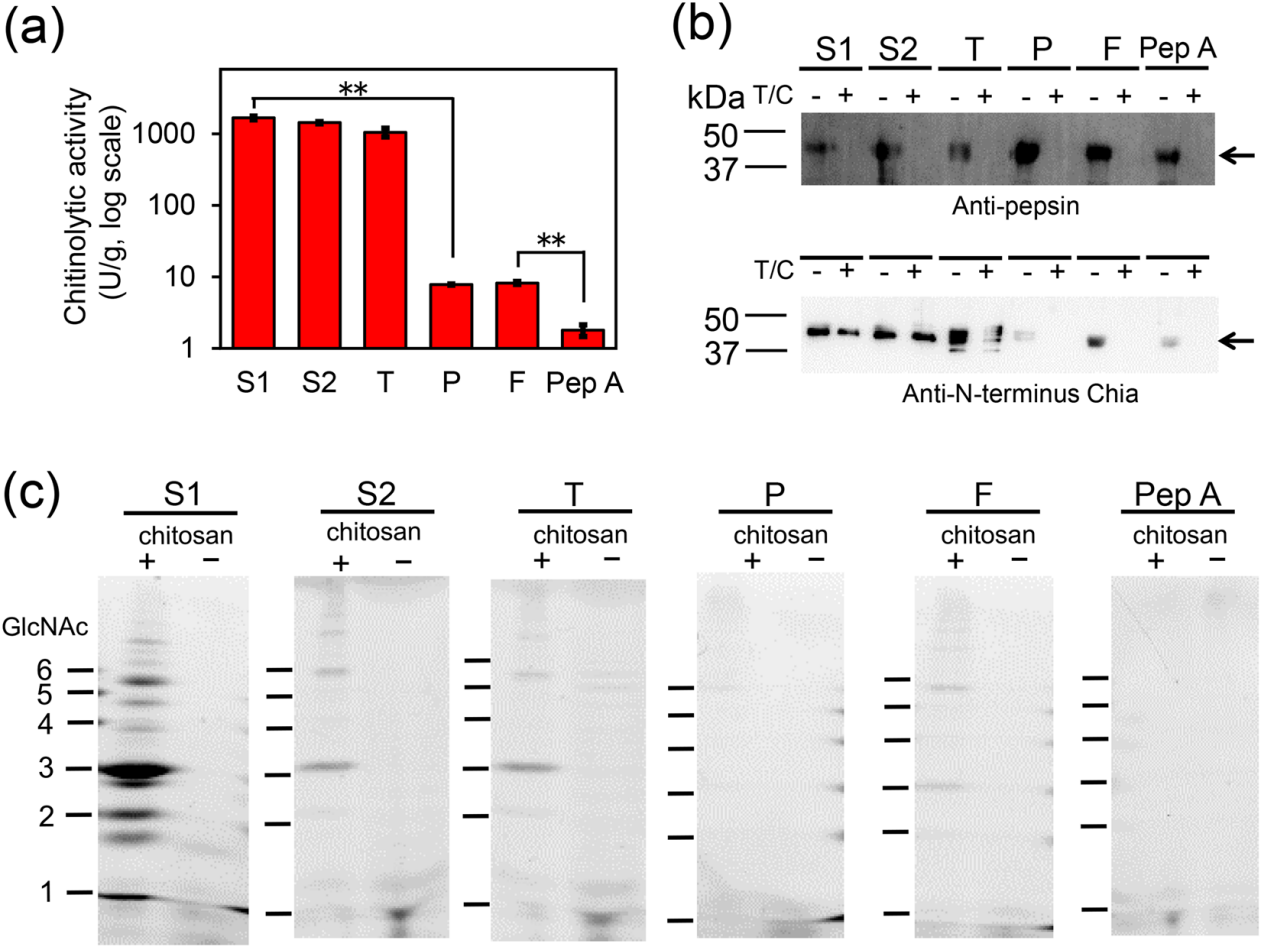
Chitinolytic and chitosan-degrading activities in six porcine pepsin preparations. (**a**) Chitinolytic activities in the five porcine pepsin preparations: P7012 (S1), P7125 (S2), T, P, F or Pep A. The panels show the logarithm of the values. Each experiment was performed in triplicate. **p < 0.01. P-values were determined using Student’s t-test. (**b**) WB using anti-porcine pepsin (upper) and anti-porcine N-terminus Chia (lower) antibodies. The images of (**b**) were cropped from dotted lines on original full-length gel images shown in Supplementary Fig. S3. (**c**) Degradation products generated by incubation of 80% DD chitosan with five pepsin preparation or purified pepsin at pH 4.0 were analyzed by FACE. The images of (**c**) were cropped from dotted lines on original full-length gel images shown in Supplementary Fig. S4a–f.

To evaluate the chitinolytic activity of truncated porcine Chia in the pepsin preparations in more detail, five porcine preparations from various manufacturers [Sigma-Aldrich P7012 (S1) and P7125 (S2)], Tokyo Chemical Industry (T), Promega (P), FUJIFILM-Wako Pure Chem (F) and Pep A were analyzed using 4-NP-(GlcNAc)_2_ as the substrate (Fig. 3a). The high chitinolytic activities were observed in the preparations of S1, S2 and T (Fig. 3a). Meanwhile, slight activity was detected from the other two preparations (P and F; activities 200 times lower than that of S1) and a minor activity was observed from Pep A, which was 900 times lower than that of S1 (Fig. 3a).

Then, the preparations and Pep A were treated by trypsin and chymotrypsin and analyzed by SDS-PAGE, followed by WB using porcine pepsin or porcine Chia antibody (Fig. 3b and Supplementary Fig. S3). The pepsin immunoreactivities were lost, however, Chia immunoreactivities were detected in the pepsin preparations of S1, S2 and T even after proteases treatment (Fig. 3b).

The tested pepsin preparations or Pep A were incubated with chitosan (DD 80%) at a final concentration of 0.5 mg/mL at pH 4.0. The degradation products were analyzed by the FACE procedure that separates and detects a very low level (pmol amount) of chitooligosaccharides^28,29^. Since FACE positive oligosaccharides were detected in the preparations of S2 and T, we removed the oligosaccharides by passing the samples through a Sephadex G-25 resin (see Supplementary Fig. S4). Preparations S1, S2, T and F degraded chitosan and produced chitooligosaccharides longer than (GlcNAc)_6_ as well as (GlcNAc)_2–6_ (Fig. 3c and Supplementary Fig. S5). On the other hand, P and Pep A did not degrade the tested substrate (Fig. 3c and Supplementary Fig. S5). Thus, chitin/chitosan-degrading activity of each pepsin preparations varied depending on the source of the truncated porcine Chia.

### Porcine Chia is physiologically processed into truncated forms by pepsin

Chia consists of the catalytic domain (CatD) and chitin-binding domain (CBD) (Supplementary Fig. S6). Mouse and chicken CatD show chitinolytic and chitin-binding activities^13,30^. To examine whether CatD can be produced under porcine stomach environment, purified porcine Chia^11,13^ was incubated with Pep A at a 1:20 ratio, which corresponds to the expression ratio of Chia and pepsin mRNA in the stomach^11^. The up-to-6 hours incubation was performed at pH 2.0 and 37 °C.

**Figure 4:**
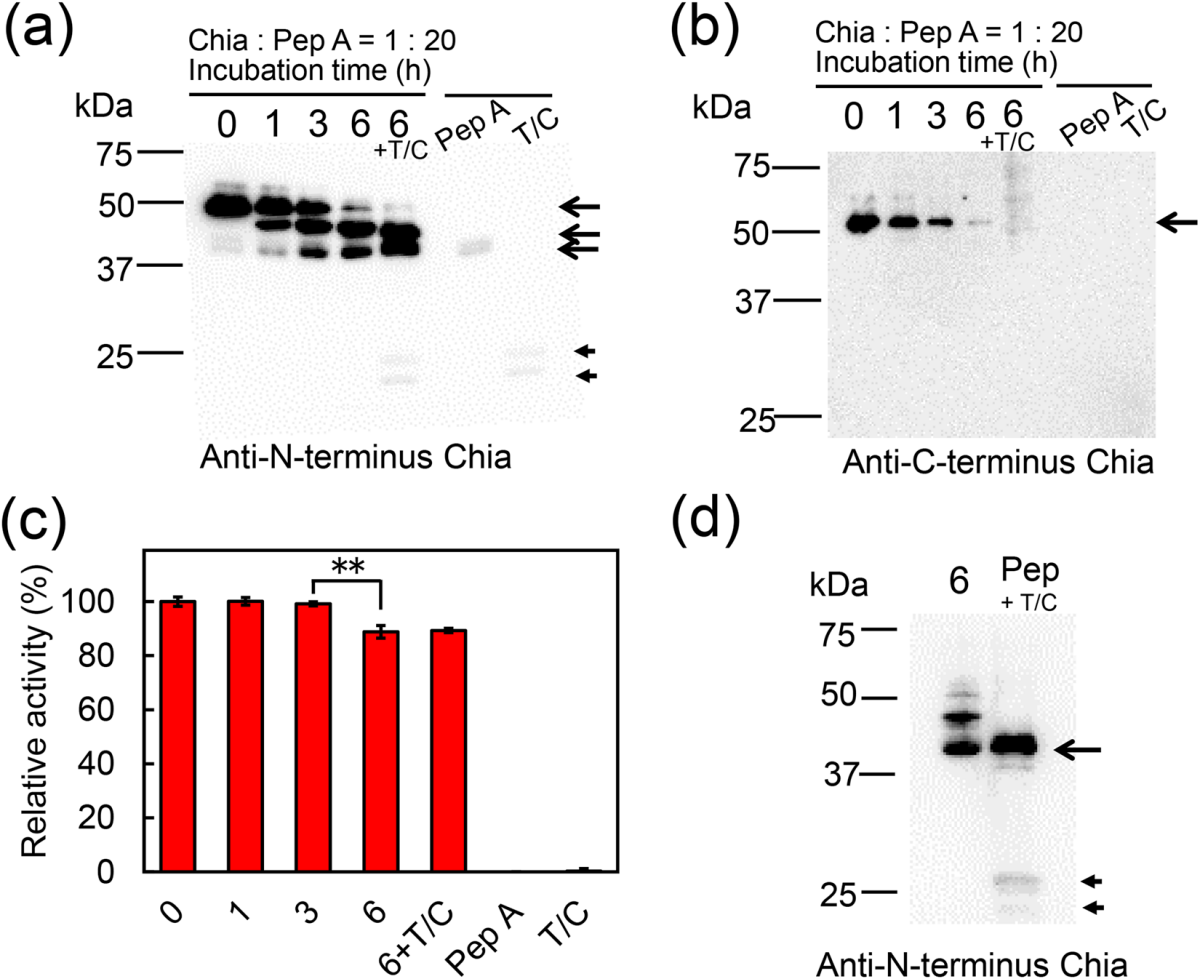
Porcine Chia is physiologically processed by pepsin. Chia protein purified from the porcine stomach was incubated with Pep A at 37 °C for 0, 1, 3, 6 hours at pH 2.0. After 6 hours-incubation, the sample was further incubated with trypsin and chymotrypsin (T/C) at pH 7.6 for 10 min. WB using (**a**) anti-pig N-terminal Chia antibody or (**b**) anti-mouse C-terminal Chia antibody. (**c**) Chitinolytic activities in the Chia without or with trypsin and chymotrypsin treatment were measured at pH 2.0 as described in Methods. Values in (**c**) represent mean ± SD from a single experiment conducted in triplicate. **p < 0.01. P-values were determined using Student’s t-test. (d) Comparison of the mobility of bands detecting by WB using anti-porcine N-terminal Chia antibody in 6-hours treated Chia by Pep A (6 + T/C) or the pepsin preparation (Pep + T/C) after trypsin/chymotrypsin treatment. The specific bands derived from trypsin/chymotrypsin were shown by arrowheads. The images of (**a**–**c**) were cropped from dotted lines on original full-length gel images shown in Supplementary Fig. S7.

As soon as after 1 hour-incubation, the full-length Chia (52 kDa) was accompanied by 40 and 45 kDa bands in WB analysis using anti-N-terminus porcine Chia antibody (Supplementary Fig. S6). After 6 hours-incubation, the full-length protein was almost not visible (Fig. 4a, lanes 0, 1, 3 and 6; Supplementary Fig. S7). Anti-C-terminus mouse Chia antibody detected only the full-length protein whose signal decreased with increasing incubation time (Fig. 4b, lanes 0, 1, 3 and 6; Supplementary Fig. S7). These results indicate that the pepsin-resistant 40 and 45 kDa band represent whole or truncated CatD, whereas CBD was degraded by Pep A.

To further evaluate the Chia stability, the protein was incubated with Pep A for 6 hours at pH 2.0, followed by incubation with trypsin and chymotrypsin at pH 7.6 for 10 min. Truncated Chia proteins were detected by anti-N-terminal Chia antibody (Fig. 4a, lane 6 + T/C) and possessed chitinolytic activity comparable to the full-length Chia (Fig. 4c and Supplementary Fig. S7).

Fu *et al*. purified three variants of chitosanases with molecular weights of 40–47 kDa from a porcine pepsin preparation^25^. Here, we show that Chia immunoreactivity in the pepsin preparation was similar in size to those of the C-terminally truncated forms of porcine Chia (Fig. 4d and Supplementary Fig. S7). These results indicate that porcine Chia was cleaved at the C-terminal end by pepsin. The CatD proteins still have excellent protease resistance and possess chitinolytic activity in the pepsin preparation (Fig. 4c). Based on our results, we suggest that the reported chitosanase variants may be due to the truncated form of Chia in the pepsin preparations.

### Characteristics of truncated forms of Chia

**Figure 5:**
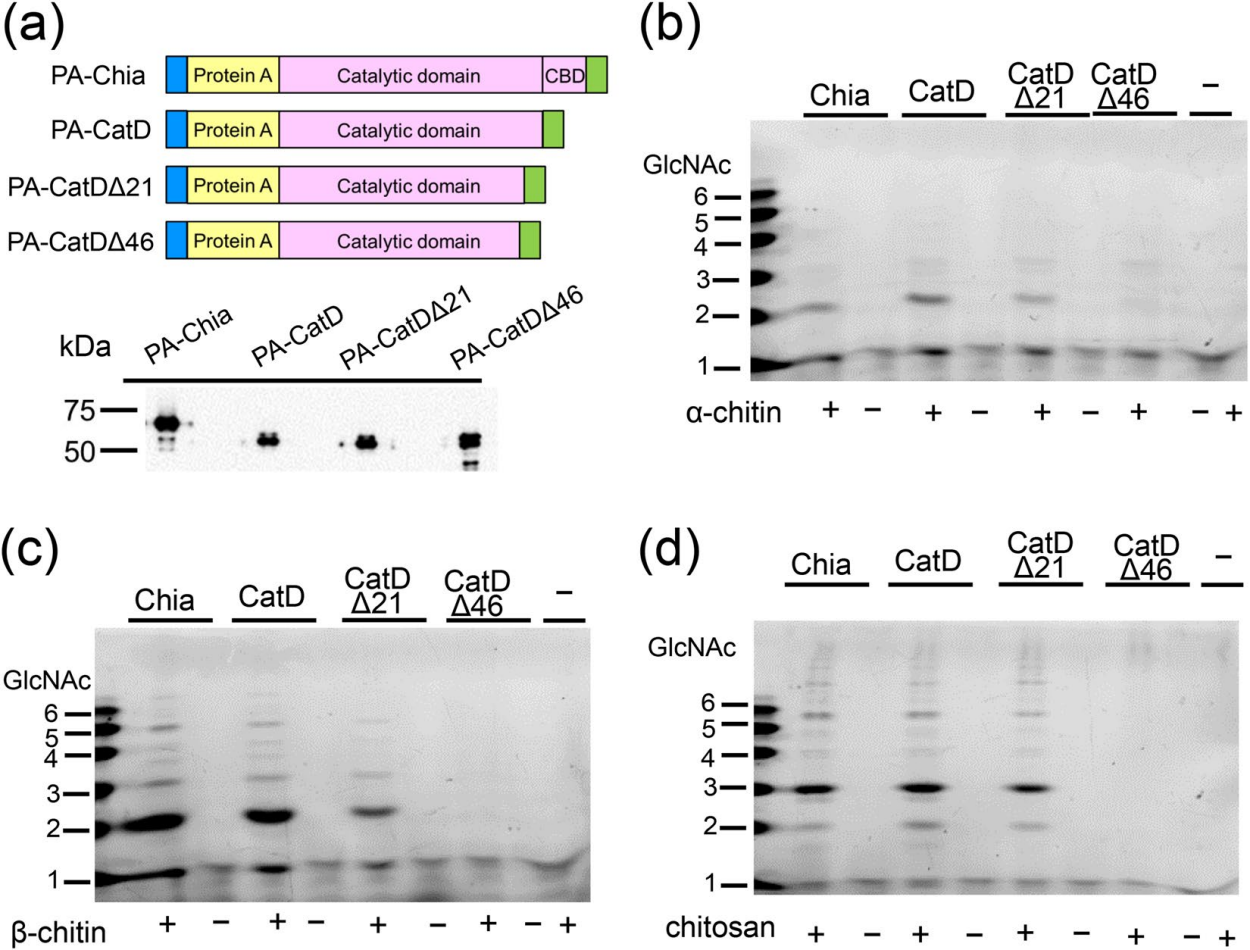
Full-length and its truncated forms of Chia possess the same chitin and chitosan degradation abilities. (**a**) The schematic representations of the recombinant fusion proteins of Protein A-full-length Chia or its truncated forms-V5-His (upper) and WB analysis of the recombinant proteins using anti-V5 antibody (lower). Degradation products generated by incubation of (**b**) α- or (**c**) β-chitin and (**d**) 80% DD chitosan with PA-Chia, PA-CatD, PA-CatDΔ24 or PA-CatDΔ46 at pH 2.0 were analyzed by FACE. The images of (**b**–**d**) were cropped dotted lines on from original full-length gel images shown in Supplementary Fig. S10.

To examine the relationship between the size of the truncated porcine Chia and the degradation activities against chitin and chitosan, the following recombinant forms of Chia proteins were constructed: CatD (pEZZ18/PA-CatD) or truncated form of CatD (pEZZ18/PA-CatDΔ21) that correspond to the 40 and 45 kDa bands in the pepsin preparation (Fig. 4a,d), and C-terminally further truncated form of CatD (pEZZ18/PA-CatDΔ46). These constructs were created according to the prediction of pepsin cleavage sites of porcine Chia using ExPASy PeptideCutter (Fig. 5a and Supplementary Figs S8 and S9).

Full-length Chia, CatD, CatDΔ21 and CatDΔ46 were expressed as recombinant fusion proteins with Protein A (PA) and V5-His (Fig. 5a) in *E*. *coli* and purified as described in Methods. The recombinant proteins were incubated first with α-chitin and degradation products were analyzed by FACE. Full-length Chia, CatD and CatDΔ21 degraded α-chitin to produce (GlcNAc)_2_ (Fig. 5b and Supplementary Fig. S10). As for β-crystalline chitin, full-length Chia, CatD and CatDΔ21 produced (GlcNAc)_2_ and (GlcNAc)_3__–__6_, while CatDΔ46 did not degrade the substrate (Fig. 5c and Supplementary Fig. S10). Full-length Chia, CatD and CatDΔ21 degraded chitosan and, besides short chitooligosaccharides, also produced (GlcNAc)_>6_ (Fig. 5d and Supplementary Fig. S10). CatDΔ46 did not affect any of the substrates. These results indicate that lack of CBD in the Chia does not inhibit its chitin- and chitosan-degrading activities, whereas further deletion of 46 amino acids from the C-terminus of the CatD caused loss of chitinolytic activity.

### Porcine Chia and its truncated forms as well as a pepsin preparation produced comparable chitooligosaccharides

Finally, the degradation products from chitosan by natural Chia or recombinant proteins and the most active pepsin preparation (S1, P7012) were compared. Several chitosan substrates differing in DD (DD 69%, 73%, 84% and 95%) were incubated with porcine Chia, its truncated forms, PA-Chia, PA-CatD or pepsin preparation as described in Methods and the products were analyzed by the FACE.

**Figure 6:**
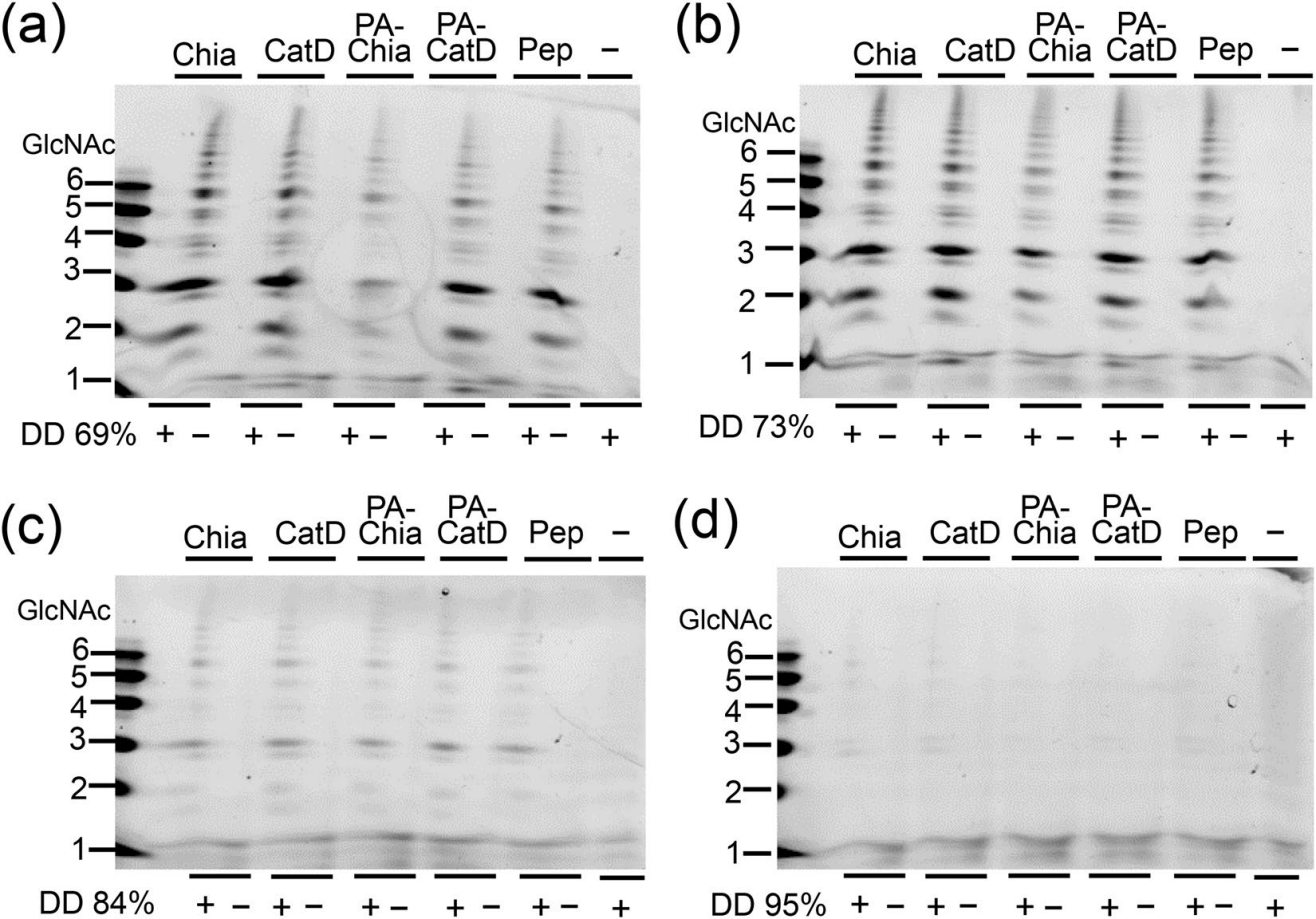
Full-length and its truncated forms of Chia as well as pepsin preparation produced comparable chitooligosaccharides. Degradation products generated by incubation of wide range of DD chitosan; (**a**) 69%, (**b**) 73%, (**c**) 84% and (**d**) 95% of DD with full-length Chia (Chia), truncated Chia (CatD), PA-Chia, PA-CatD or the pepsin preparation (Pep) at pH 4.0 were analyzed by FACE. The images of (**a**–**d**) were cropped from dotted lines on original full-length gel images shown in Supplementary Fig. S11.

Chitosan substrates with DD of 69%, 73% and 84% were degraded to up to (GlcNAc)_~20_. The degradation pattern was similar for all enzymes (Fig. 6a–d and Supplementary Fig. S11). The digestion efficiency decreased with increasing DD of chitosan and only a very limited degradation was observed in DD 95% (Fig. 6a–d). These results indicate that chitosan-degrading activity in the pepsin preparations is due to the chitinolytic activity of truncated Chia which can digest chitosan.

Furthermore, the ability of porcine Chia and the pepsin preparation to degrade (GlcNAc)_5_ as well as (GlcN)_5_ and (GlcN)_6_ was investigated. None of the GlcN substrates was digested, while (GlcNAc)_5_ was completely degraded into (GlcNAc)_2_ and GlcNAc monomer (Fig. S12). These results indicate that the chitosan-degrading activity of porcine Chia and the pepsin preparation appears to be mediated by chitinolytic rather than by *per se* chitosanolytic activity with no effect on GlcN-GlcN bonds.

## Discussion

In this study, we investigated the chitosan-degrading (chitosanase) activity in the commercially available porcine pepsin preparations, which have been used for enzymatic production of chitooligosaccharides. We detected residual active fragments (CatD) derived from Chia displaying chitosan-degrading activity similar to those of the full-length and C-terminally truncated porcine Chia.

Functional properties of recombinant mouse CatD are comparable with those of full-length Chia^30^. Natural porcine CatD, obtained by incubation of Chia with purified pepsin, showed a comparable degradation activity against chitin and chitosan by the full-length enzyme. This activity is present even in a further truncated form of CatD by up to 24 amino acids, while the deletion of 46 amino acids leads to activity loss. These results suggested that at least five of the six cysteines conserved in the catalytic domain^31^ are sufficient to keep the proper tertiary structure required for chitinolytic activity and/or chitin/chitosan substrates recognition.

Porcine Chia and pepsin preparation degraded chitosan substrates with DD of 69–84%. However, the digestion efficiency decreased with increasing DD and only a very limited degradation was observed in DD of 95% chitosan suggesting the absence of a *per se* “chitosanase” activity of Chia. To investigate the mechanism of chitosan degradation by porcine Chia, (GlcNAc)_5_ as well as (GlcN)_5_ and (GlcN)_6_ were exposed to the enzyme (Fig. S12). While (GlcNAc)_5_ was completely digested into (GlcNAc)_2_ and GlcNAc monomer, the GlcN substrates remained stable (Fig. S12). These results indicate that porcine Chia is not able to hydrolyze GlcN-GlcN bonds, unlike chitosanases^32^ and that the chitosan-degrading activity in the pepsin preparations is due to its chitinolytic activity.

Porcine Chia degraded chitin and (GlcNAc)_5_ producing mainly (GlcNAc)_2_ (Fig. 1a) and (GlcNAc)_2_ and GlcNAc monomer (Fig. S12), respectively. When chitosan was treated with porcine Chia or pepsin preparations, the main products with mobility similar to (GlcNAc)_3_ were observed (Figs 1a, 3c, 5d, 6a–c and Fig. S13). Intuitively, hydrolysis of (GlcNAc)_3_ into (GlcNAc)_2_ and GlcNAc monomer was expected. While it is difficult to explain the stability of the major product, the following can be considered: *Serratia marcescens* and *Streptomyces coelicolor* chitinases can degrade chitosan and sequentially produce hetero-chitotrimers, such as GlcN-GlcNAc-GlcNAc and GlcNAc-GlcN-GlcNAc, as revealed by Nuclear Magnetic Resonance (NMR) spectroscopy^33–37^. Similar results were also obtained with the chitosan degradation by human chitotriosidase (Chit1)^38^. Thus, the main degradation products of chitosan treated by porcine Chia or the pepsin preparations may possibly be hetero-chitotrimers. Moreover, a minor band below the main product was observed (Figs 1a, 3c, 5d and 6a–c), suggesting the presence of at least one hetero-chitotrimer within the trimer products. Importantly, two bands were also detected at each size of the (GlcN)_1–6_ standard labeled by our improved FACE method^29^ (right margin in Fig. S13a,b). The presence of multiple bands in the chitosan and GlcN oligomer size markers has been shown previously^10^. This event warrants further investigation pursued by our group.

The presence of heterogenic products might be delineated by the utilized chitosan structure. The fact that we used heterogeneously deacetylated substrates with highly deacetylated areas in the amorphous region as compared to the crystalline section^39^ suggests that (GlcNAc)_2_ or (GlcNAc/GlcN)_3_ is generated from the crystalline region while the longer fragments such as (GlcNAc/GlcN)_>6_ result from the non-crystalline part of the substrate. The hypothesis of the major band only consisting of hetero-chitotrimers will have to be confirmed once the hetero-chitotrimer standards are available. Further studies on the specificity of the oligomers produced from chitosan by porcine Chia as well as on the digestion mechanism and site-binding preferences of the enzyme will be needed for enhancing its application for the production of well-defined chitooligosaccharides.

Interestingly, chitosan-degrading activities have also been found in plant-derived papain^40^ and bacteria-derived pronase^23^. The data presented here suggest that such chitosan-degrading activities may result from “contamination” of the respective preparations by protease-resistant chitosan-degrading enzymes rather than from the intrinsic properties of these proteases. Plants have been known to synthesize various types of chitinases for protection from chitin-containing pathogens. A thorough evaluation of papain and pronase preparations including a detailed biochemical characterization of chitosan-degrading activity may even lead to the discovery of novel forms of chitinases or other enzymes with such activity.

Since purified chitosanase is an expensive material, many enzymes with different original specificities have been evaluated for their ability to hydrolyze chitosan^40–42^. According to our knowledge, only few mammalian enzymes can degrade chitosan. Thus, the use of natural porcine Chia or chitosan-degrading activity present in pepsin preparations may facilitate the production of chitooligosaccharides that are useful in the biochemical and food industry.

## Conclusion

The commercially available pepsin preparations can degrade chitosan into chitooligosaccharides. We detected protease-resistant truncated Chia responsible for the chitinolytic activity in the pepsin preparations. Chia and its pepsin-truncated forms as well as the pepsin preparations did not have chitosanolytic activity but degraded DD 69–84% chitosan substrates with comparable efficiency. Thus, the chitosan degrading activity in the porcine pepsin preparations is due to chitinolytic activity of truncated forms of Chia. Porcine Chia present in the pepsin preparations can be used for the production of chitooligosaccharides from chitosan substrates.

## Methods

### Pepsin preparations used in this study

Pepsin preparations were obtained from Sigma-Aldrich (P7012 and P7125) (St. Louis, MO, USA), Tokyo Chemical Industry Co. (9001–75–6) (Tokyo, Japan), Promega (V1959) (Madison, WI, USA) and FUJIFILM-Wako Pure Chemical Co. (165–18711) (Osaka, Japan). Purified pepsin A (Pep A, two times crystallized. LS003319) was purchased from Worthington Biochemical Co. (Lakewood, NJ, USA).

### Degradation of chitosan and α- or β-crystalline chitin substrates by pepsin preparations

Chitosan [degree of deacetylation (DD) 80%, Chitosan 100] and α-chitin from shrimp shell chitin were purchased from FUJIFILM-Wako Pure Chem. Co. and Sigma-Aldrich, respectively. β-chitin from squid pens was a generous gift from Katakura & Co-op Agri Corporation (Tokyo, Japan). DD was determined by elemental analysis as described previously^20^. Chitosan, α- or β-crystalline chitin substrate (1 mg/mL) was incubated in a volume of 50 µL containing pepsin preparation (P7012, Sigma-Aldrich) (0.5 mg/mL) or Pep A (0.5 mg/mL) in McIlvaine’s buffer (pH 4.0) at 37 °C for 16 hours. Generated chitin fragments were analyzed by FACE^28,29^.

### Chitinase enzymatic assays

The chitinolytic activity was determined using a synthetic substrate, 4-nitrophenyl *N*,*N’*-diacetyl-β-D-chitobioside [4-NP-(GlcNAc)_2_, Sigma-Aldrich] essentially as described previously^43^. One enzyme unit (U) was defined as 1 μmol of 4-nitrophenol released from 4-NP-(GlcNAc)_2_ per min at 37 °C in Gly-HCl buffer (pH 2.0). All enzymatic reactions for optimum pH and temperature determination were conducted in a volume of 50 μL as described previously^30,43^.

For determination of the optimal pH, the chitinase activity was evaluated by incubating the enzyme with the 4-NP-(GlcNAc)_2_ substrate in 0.1 M Gly-HCl buffer (pH 1.0–3.0) or McIlvaine’s buffer (0.1 M citric acid and 0.2 M Na_2_HPO_4_; pH 2.0–8.0) at 37 °C for 30 min. To determine the optimal temperature, chitinase activity was assayed between 30 °C and 64 °C in 0.1 M Gly-HCl buffer (pH 2.0).

### SDS-polyacrylamide gel electrophoresis (PAGE) and WB

The obtained protein fractions were analyzed using standard SDS-PAGE, followed by Coomassie Brilliant Blue R-250 (CBB, Sigma-Aldrich) or WB using anti-porcine N-terminal Chia (rabbit)^12^ anti-mouse C-terminal Chia (rabbit)^44^ or anti-porcine pepsin antibody (donkey) (GeneTex, Irvine, CA, USA), followed by peroxidase-conjugated AffiniPure F (ab’)_2_ Fragment Donkey Anti-Rabbit IgG (H + L) (Jackson ImmunoResearch Laboratories, Inc., West Grove, PA, USA) or AffiniPure Donkey Anti-Goat IgG-HRP (Jackson ImmunoResearch laboratories). The immunoblots were analyzed by Luminescent Image Analyzer (ImageQuant LAS 4000, GE Healthcare, Piscataway, NJ, USA) according to the manufacturer’s instructions.

### Pepsin enzymatic assays

Proteolytic activity of the pepsin preparation was measured using hemoglobin from bovine blood (Sigma-Aldrich) as the substrate as described previously^9^.

### Degradation of chitosan by pepsin preparations or Pep A

Chitosan substrate (1 mg/mL) was incubated in a volume of 50 µL containing pepsin preparations or Pep A (0.5 mg/mL) in McIlvaine’s buffer (pH 4.0) at 37 °C for 16 hours. Generated chitin fragments were analyzed as described above.

### Porcine stomach tissues

Six months-old male porcine stomach tissues (Landrace F1) were purchased from Funakoshi Co., Ltd (Tokyo, Japan), which were dissected from the animals, quickly frozen on dry ice and kept at −80 °C.

### Purification of porcine Chia

The natural Chia enzyme was purified from porcine stomach tissue using chitin beads column and eluted with 0.1 M acetic acid as described previously^13^. The eluted enzymes were neutralized and desalted with PD10 (GE Healthcare) equilibrated by TS buffer.

Protein concentrations were determined by the Bradford Protein Assay (Bio-Rad Laboratories, Hercules, CA, USA) using BioPhotometer Plus UV/Vis equipment (Eppendorf, Hamburg, Germany). Bovine serum albumin was used as the standard.

### Detection of truncated Chia in pepsin preparations

To investigate the presence of porcine Chia in the tested pepsin preparations, the preparations or Pep A (0.5 µg) were incubated with trypsin and chymotrypsin (0.5 µg) (Sigma-Aldrich) at pH 7.6 for 10 min. After the incubation, a protease inhibitor (Complete Mini, Roche, Basel, Switzerland) was added and analyzed by WB as described above.

### Preparation of recombinant porcine Chia and truncated porcine Chia proteins

The full-length, catalytic domain (CatD) or truncated CatD of porcine Chia were expressed as recombinant fusion proteins with Protein A (PA) and V5-His. The pEZZ18/PA-porcine Chia-V5-His was prepared as described previously^45^. The regions of interest were amplified from the porcine full-length Chia-expressing plasmid DNA (pEZZ18/pre-PA-porcine Chia-V5-His) using oligonucleotide primers (Supplementary Table S1) as described previously^30^. Each amplified DNA was then digested with EcoRI and XhoI and subcloned into the pEZZ18 expression vector. The entire nucleotide sequence of the resulting plasmid DNAs (pEZZ18/PA-CatD, pEZZ18/PA-CatDΔ21 or PA-CatDΔ46) was confirmed by sequencing (Eurofins Genomics, Tokyo, Japan). The recombinant PA-Chia, PA-CatD, PA-CatDΔ21 and PA-CatDΔ46 (Supplementary Fig. S9) were prepared as described previously^30,45^.

### Degradation of chitosan with different DD by Chia and its truncated forms as well as pepsin preparation

Heterogeneously deacetylated chitosan (block-type chitosan)^39^ with DD 69%, 73%, 84% and 95% were generous gifts from Funakoshi Co., Ltd. The chitosan substrates (1 mg/mL) were incubated in a volume of 50 μL containing full-length or truncated Chia, PA-Chia, PA-CatD or pepsin preparation (50 µU) as described previously^11^. Generated chitin fragments were analyzed by FACE^28,29^. *N*-acetyl chitooligosaccharides (Seikagaku Corporation, Tokyo, Japan) were used as a standard.

### Statistical analysis

Biochemical data were compared by Student’s t-test. We carried out experiments in triplicate for the statistical analysis.

## Supplementary information

Supporting Information

## Data Availability

The datasets generated and/or analyzed during the current study are available from the corresponding author on reasonable request.
