# Supplementary material for: Residues of acidic chitinase cause chitinolytic activity degrading chitosan in porcine pepsin preparations
Source: Sci Rep. 2019 Oct 30;9:15609. doi: 10.1038/s41598-019-52136-2 (PMC6821832; doi:10.1038/s41598-019-52136-2)
Supplement: Supplementary file 1 — Supporting Information [file 41598_2019_52136_MOESM1_ESM.pdf]

Residues of acidic chitinase cause chitinolytic activity degrading chitosan in porcine pepsin preparations

Eri Tabata[1,2], Satoshi Wakita[1], Akinori Kashimura[1], Yasusato Sugahara[1], Vaclav Matoska[3], Peter O. Bauer[3,4] & Fumitaka Oyama[1]

[1]Department of Chemistry and Life Science, Kogakuin University, Hachioji, Tokyo 192-0015, Japan, [2]Research Fellow of Japan Society for the Promotion of Science (DC1), Koujimachi, Chiyoda-ku, Tokyo 102-0083, Japan, [3]Laboratory of Molecular Diagnostics, Department of Clinical Biochemistry, Hematology and Immunology, Homolka Hospital, Roentgenova 37/2, Prague 150 00, Czech Republic, [4]Bioinova Ltd., Videnska 1083, Prague 142 20, Czech Republic

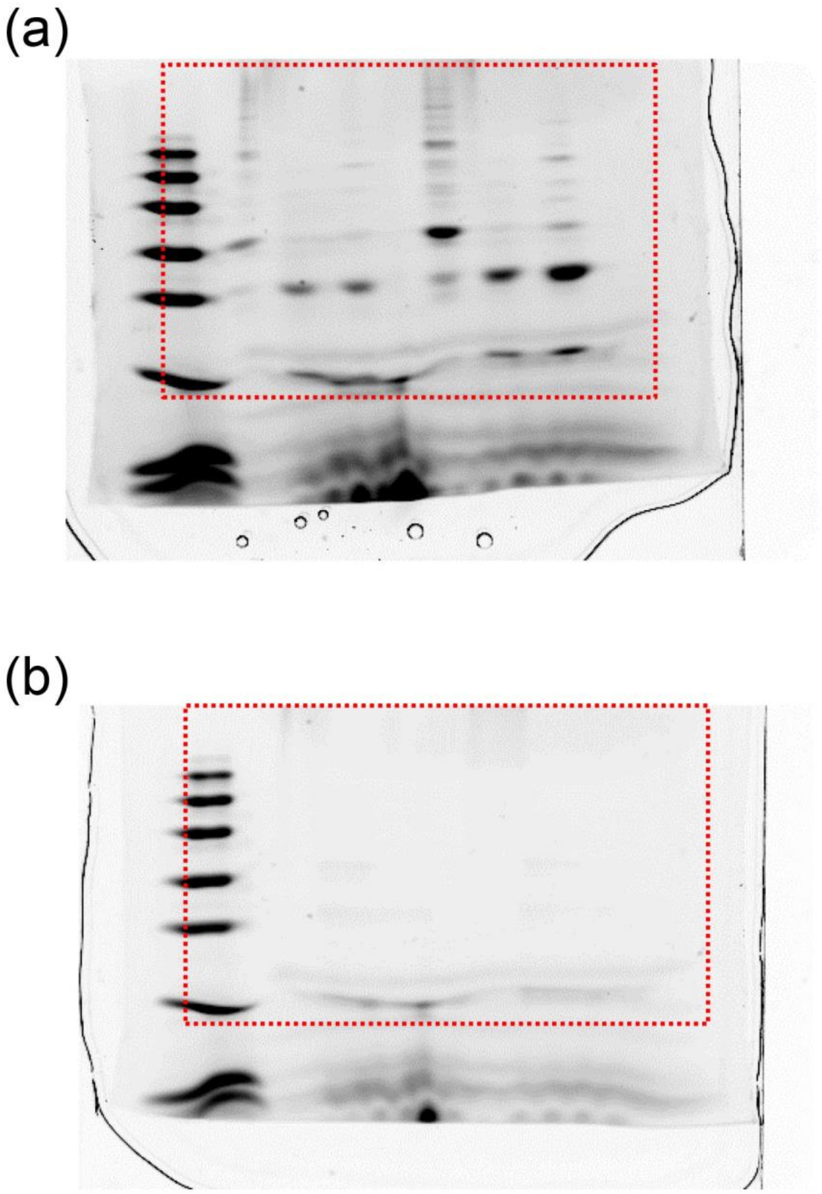

Supplementary Figure S1. Full-length gel and blots shown in Fig. 1. (a and b) Full-length gel images of the FACE methods in Fig. 1a and b.

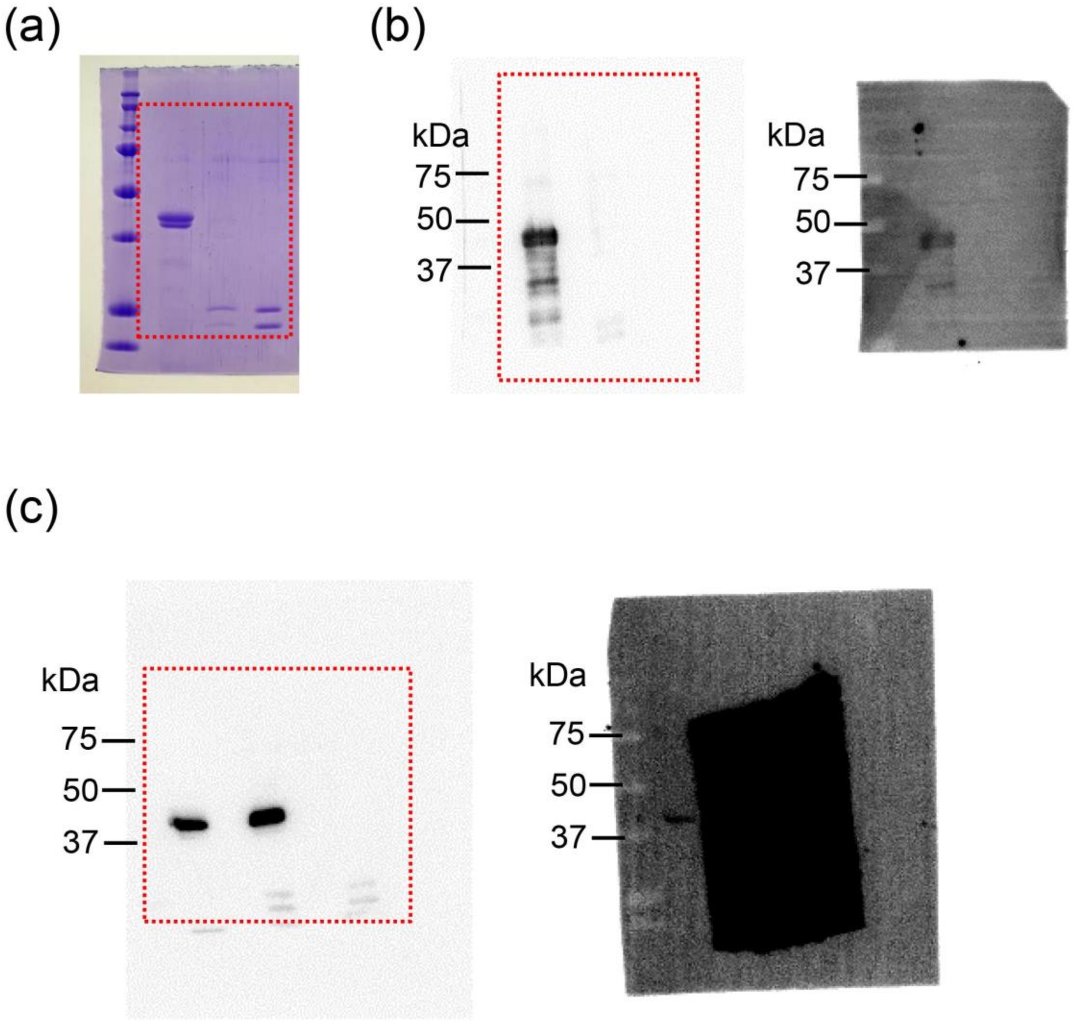

Supplementary Figure S2. Full-length gel and blots shown in Fig. 2. (a) Full-length gel images of SDS-PAGE and CBB staining. (b) Western blotting using anti-pepsin antibody. (c) Western blotting using anti-N-terminus Chia antibody.

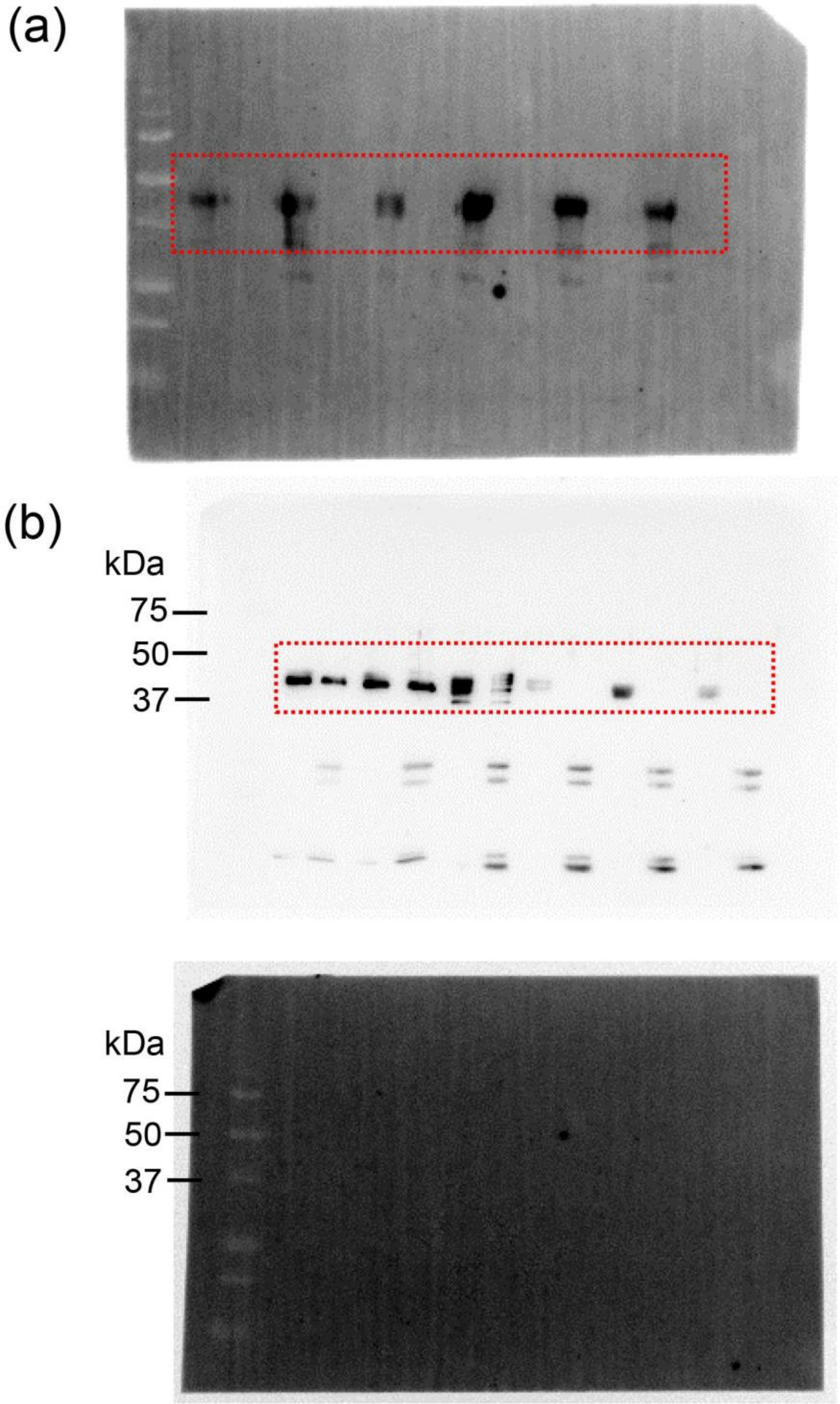

Supplementary Figure S3. Full-length gel and blots shown in Fig. 3. (a and b) Western blotting using anti-pepsin antibody and anti-N-terminus Chia antibody, respectively, in Fig. 3b.

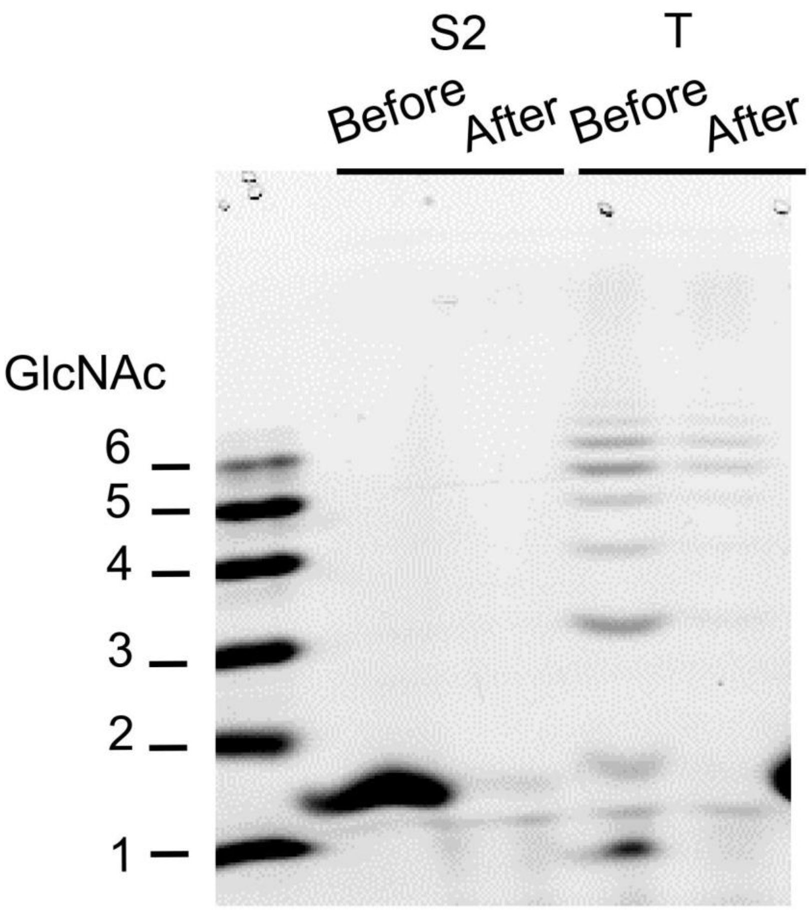

Supplementary Figure S4. Detection of FACE positive oligosaccharides in the pepsin preparation and their removal. To remove the carbohydrates derived from the preparations of Sigma-Aldrich (S2, P7125) and Tokyo Chemical Industry (T) (Fig. 3c), the preparations were incubated in McIlvaine's buffer (pH 4.0) at 37°C for 16 hours and applied onto PD MiniTrap G-25 (GE Healthcare) equilibrated with TS buffer [20 mM Tris-HCl, 150 mM NaCl (pH 7.6)] and used for subsequent analysis. After the incubation, FACE positive oligosaccharides were disappeared.

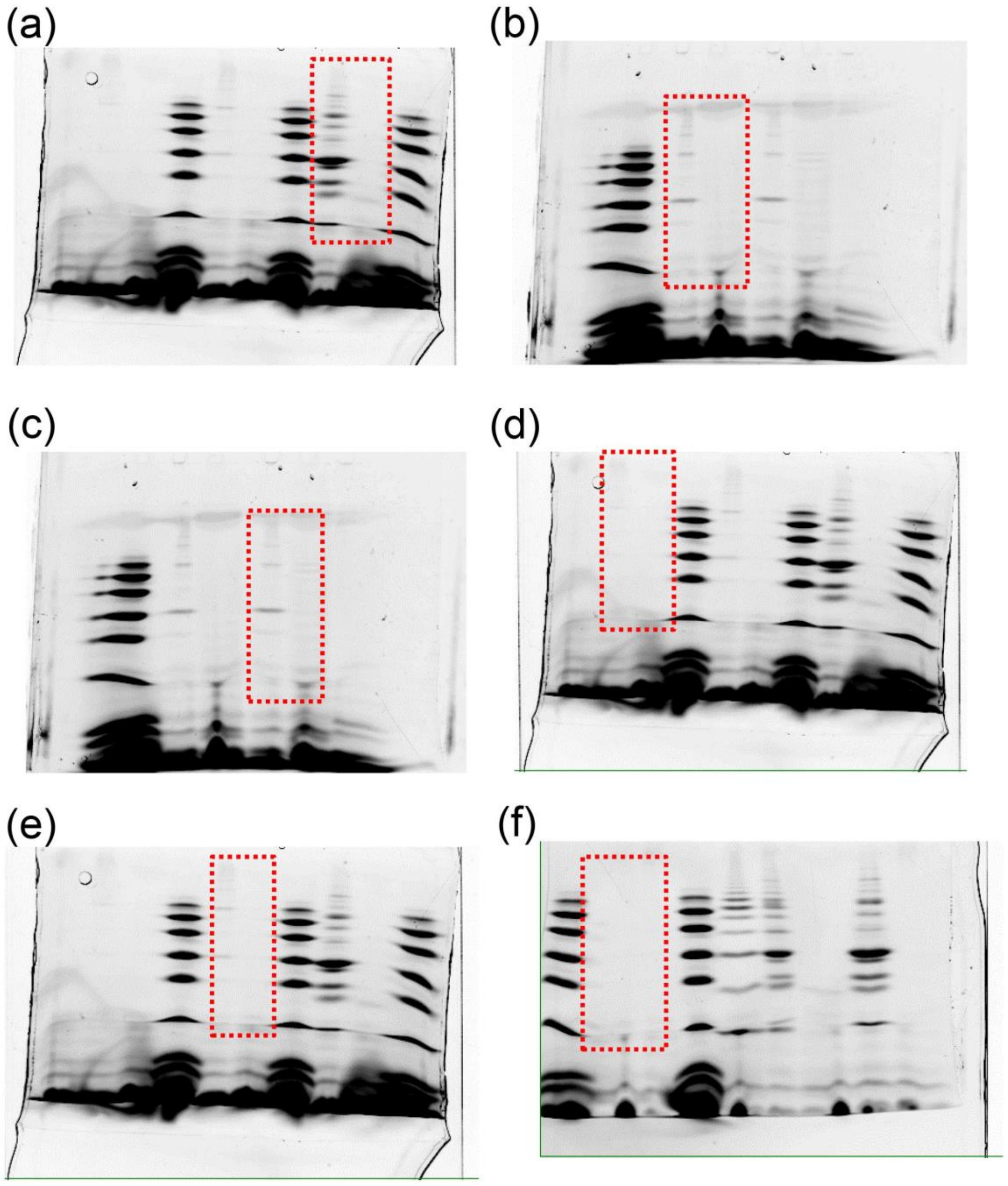

Supplementary Figure S5. Full-length gel and blots shown in Fig. 3. (a-f) Full-length gel images of the FACE methods in Fig. 3c. (a) P7012 (S1); (b) P7125 (S2); (c) T; (d) P; (e) F; (f) Pep A.

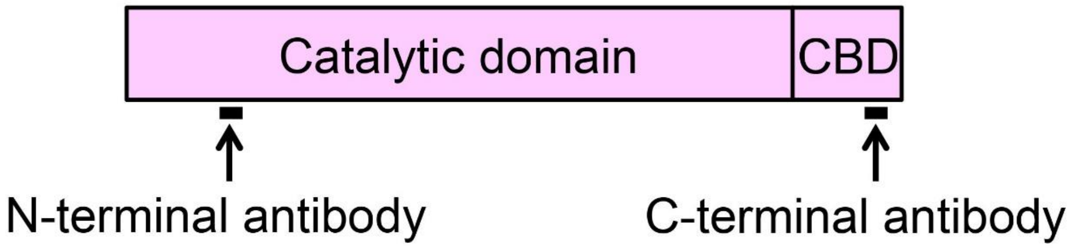

Supplementary Figure S6. Schematic representation of domain structure porcine Chia and location of antigenic sites. Porcine Chia is composed of an N-terminal CatD and a C-terminal CBD. We established specific antibodies against porcine Chia. The locations of antigen sites for N- and C-terminal antibodies used in the study are shown.

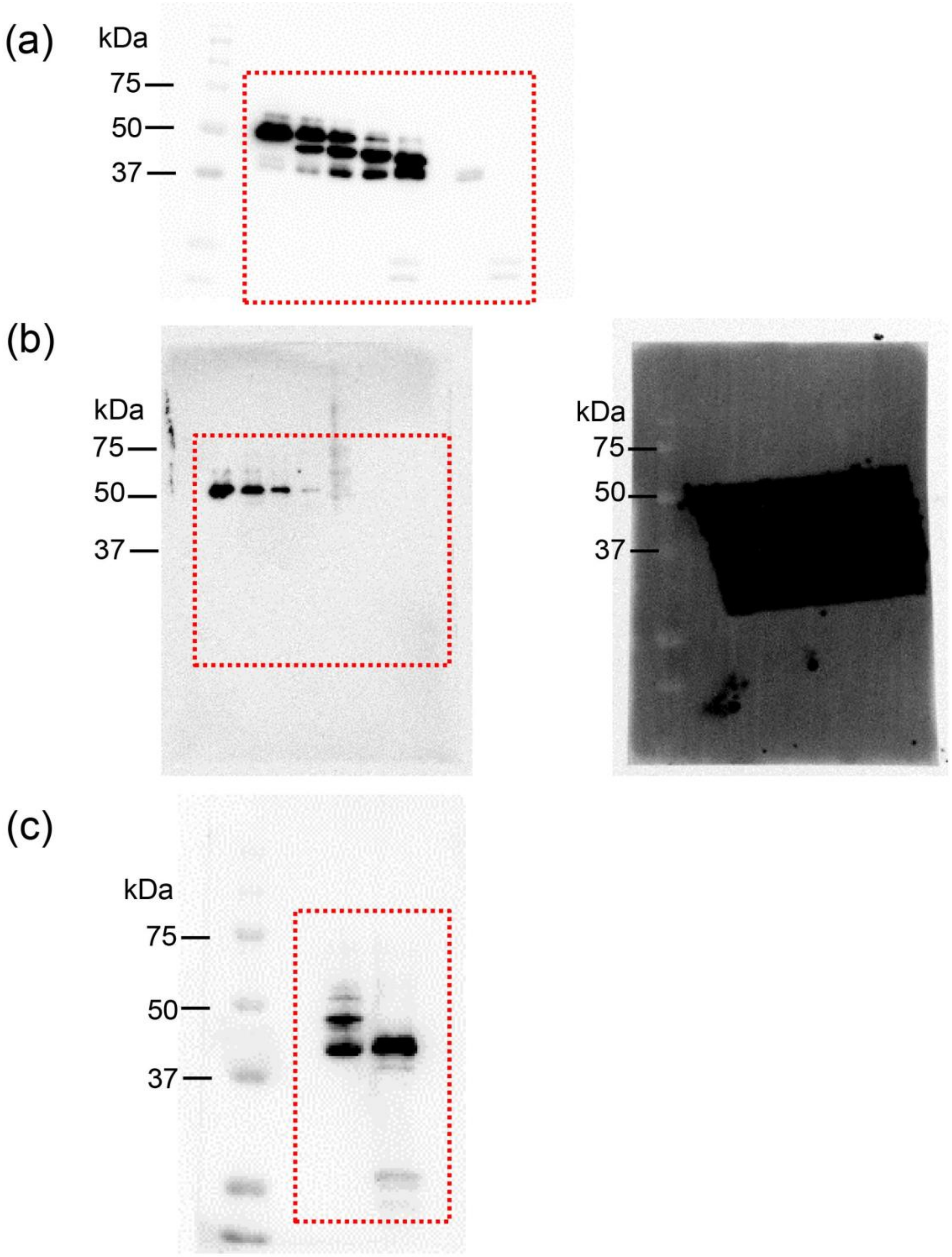

Supplementary Figure S7. Full-length blots shown in Fig. 4. (a-c) Western blotting using anti-N-terminus Chia antibody, anti-C-terminus Chia or anti-N-terminus Chia shown in Fig. 4a, b or d, respectively.

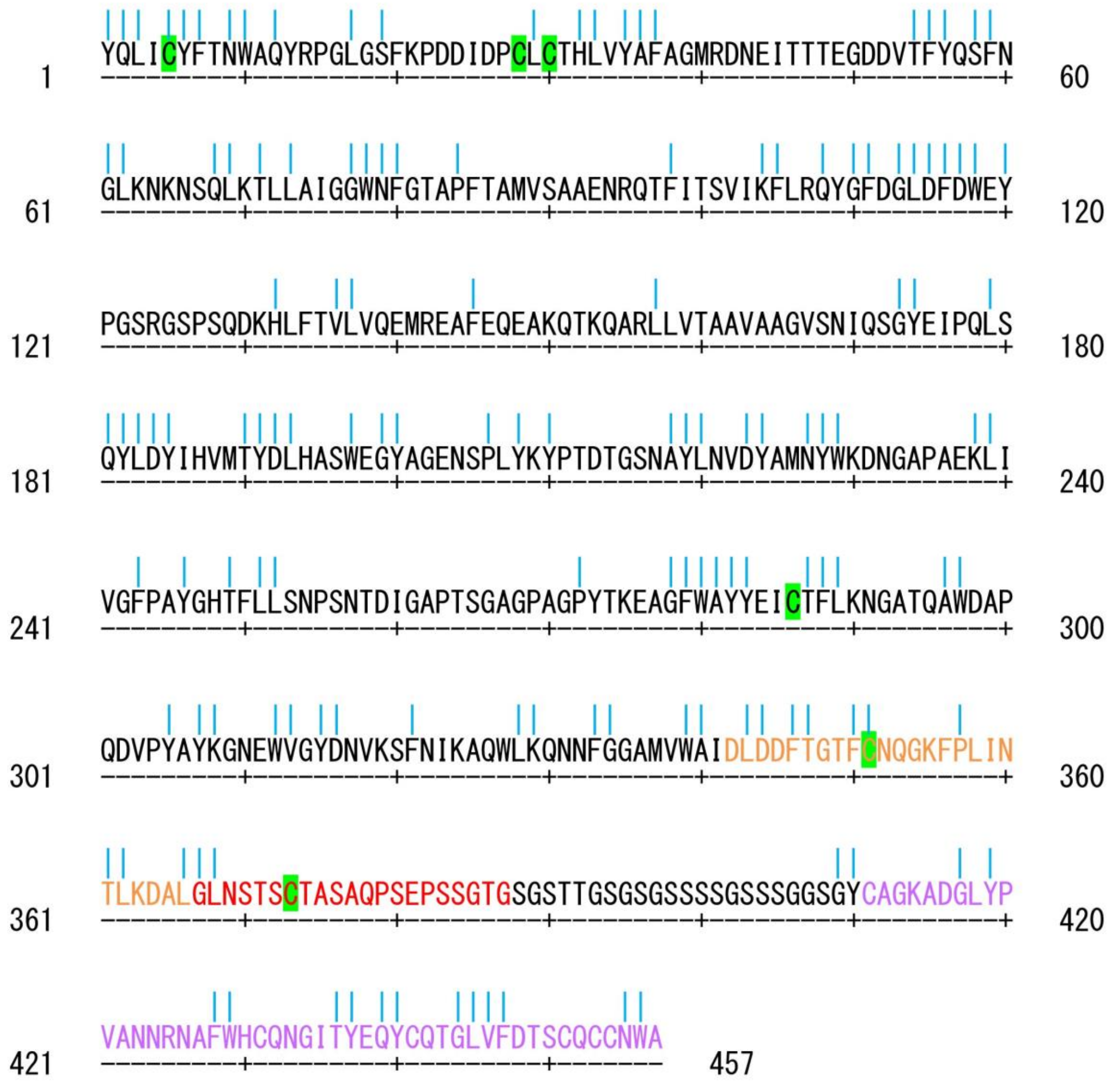

Supplementary Figure S8. Potential Pepsin cleavage sites by Pepsin digestion. Pepsin cleavage sites of porcine Chia predicted by ExPASy PeptideCutter (https://web.expasy.org/peptide_cutter/) are shown in light blue. Cysteine residues are highlighted by green color. CBD region is shown in purple. In addition to CatD, we expressed truncated CatD proteins with further 21 amino acid deletion (shown in red) and 46 amino acid deletion (shown in red and orange) as a recombinant fusion protein.

```
Full length   MKKKNIYSIRKLGVGIASVTLGTLLISGGVTPAANAAQHDEAVDNKFNKEQQNAFYEILH   60
CatD          MKKKNIYSIRKLGVGIASVTLGTLLISGGVTPAANAAQHDEAVDNKFNKEQQNAFYEILH   60
CatDΔ21       MKKKNIYSIRKLGVGIASVTLGTLLISGGVTPAANAAQHDEAVDNKFNKEQQNAFYEILH   60
CatDΔ46       MKKKNIYSIRKLGVGIASVTLGTLLISGGVTPAANAAQHDEAVDNKFNKEQQNAFYEILH   60

Full length   LPNLNEEQRNAFIQSLKDDPSQSANLLAEAKKLNDAQAPKVDNKFNKEQQNAFYEILHLP   120
CatD          LPNLNEEQRNAFIQSLKDDPSQSANLLAEAKKLNDAQAPKVDNKFNKEQQNAFYEILHLP   120
CatDΔ21       LPNLNEEQRNAFIQSLKDDPSQSANLLAEAKKLNDAQAPKVDNKFNKEQQNAFYEILHLP   120
CatDΔ46       LPNLNEEQRNAFIQSLKDDPSQSANLLAEAKKLNDAQAPKVDNKFNKEQQNAFYEILHLP   120

Full length   NLNEEQRNAFIQSLKDDPSQSANLLAEAKKLNDAQAPKVDANSYQLICYFTNWAQYRPGL   180
CatD          NLNEEQRNAFIQSLKDDPSQSANLLAEAKKLNDAQAPKVDANSYQLICYFTNWAQYRPGL   180
CatDΔ21       NLNEEQRNAFIQSLKDDPSQSANLLAEAKKLNDAQAPKVDANSYQLICYFTNWAQYRPGL   180
CatDΔ46       NLNEEQRNAFIQSLKDDPSQSANLLAEAKKLNDAQAPKVDANSYQLICYFTNWAQYRPGL   180

Full length   GSFKPDDIDPCLCTHLVYAFAGMRDNEITTTEGDDVTFYQSFNGLKNKNSQLKTLLAIGG   240
CatD          GSFKPDDIDPCLCTHLVYAFAGMRDNEITTTEGDDVTFYQSFNGLKNKNSQLKTLLAIGG   240
CatDΔ21       GSFKPDDIDPCLCTHLVYAFAGMRDNEITTTEGDDVTFYQSFNGLKNKNSQLKTLLAIGG   240
CatDΔ46       GSFKPDDIDPCLCTHLVYAFAGMRDNEITTTEGDDVTFYQSFNGLKNKNSQLKTLLAIGG   240

Full length   WNFGTAPFTAMVSAAENRQTFITSVIKFLRQYGFDGLDFDWEYPGSRGSPSQDKHLFTVL   300
CatD          WNFGTAPFTAMVSAAENRQTFITSVIKFLRQYGFDGLDFDWEYPGSRGSPSQDKHLFTVL   300
CatDΔ21       WNFGTAPFTAMVSAAENRQTFITSVIKFLRQYGFDGLDFDWEYPGSRGSPSQDKHLFTVL   300
CatDΔ46       WNFGTAPFTAMVSAAENRQTFITSVIKFLRQYGFDGLDFDWEYPGSRGSPSQDKHLFTVL   300

Full length   VQEMREAFEQEAKQTKQARLLVTAAVAAGVSNIQSGYEIPQLSQYLDYIHVMTYDLHASW   360
CatD          VQEMREAFEQEAKQTKQARLLVTAAVAAGVSNIQSGYEIPQLSQYLDYIHVMTYDLHASW   360
CatDΔ21       VQEMREAFEQEAKQTKQARLLVTAAVAAGVSNIQSGYEIPQLSQYLDYIHVMTYDLHASW   360
CatDΔ46       VQEMREAFEQEAKQTKQARLLVTAAVAAGVSNIQSGYEIPQLSQYLDYIHVMTYDLHASW   360

Full length   EGYAGENSPLYKYPTDTGSNAYLNVDYAMNYWKDNGAPAEKLIVGFPAYGHTFLLSNPSN   420
CatD          EGYAGENSPLYKYPTDTGSNAYLNVDYAMNYWKDNGAPAEKLIVGFPAYGHTFLLSNPSN   420
CatDΔ21       EGYAGENSPLYKYPTDTGSNAYLNVDYAMNYWKDNGAPAEKLIVGFPAYGHTFLLSNPSN   420
CatDΔ46       EGYAGENSPLYKYPTDTGSNAYLNVDYAMNYWKDNGAPAEKLIVGFPAYGHTFLLSNPSN   420

Full length   TDIGAPTSGAGPAGPYTKEAGFWAYYEICTFLKNGATQAWDAPQDVPYAYKGNEWVGYDN   480
CatD          TDIGAPTSGAGPAGPYTKEAGFWAYYEICTFLKNGATQAWDAPQDVPYAYKGNEWVGYDN   480
CatDΔ21       TDIGAPTSGAGPAGPYTKEAGFWAYYEICTFLKNGATQAWDAPQDVPYAYKGNEWVGYDN   480
CatDΔ46       TDIGAPTSGAGPAGPYTKEAGFWAYYEICTFLKNGATQAWDAPQDVPYAYKGNEWVGYDN   480

Full length   VKSFNIKAQWLKQNNFGGAMVWAIDLDDFTGTFCNQGKFPLINTLKDALGLNSTSCTASA   540
CatD          VKSFNIKAQWLKQNNFGGAMVWAIDLDDFTGTFCNQGKFPLINTLKDALGLNSTSCTASA   540
CatDΔ21       VKSFNIKAQWLKQNNFGGAMVWAIDLDDFTGTFCNQGKFPLINTLKDAL-----------   529
CatDΔ46       VKSFNIKAQWLKQNNFGGAMVWAI------------------------------------   504

Full length   QPSEPSSGTGSGSTTGSGSGSSSSGSSSGGSGYCAGKADGLYPVANNRNAFWHCQNGITY   600
CatD          QPSEPSSGTG--------------------------------------------------   550
CatDΔ21       ------------------------------------------------------------   529
CatDΔ46       ------------------------------------------------------------   504

Full length   EQYCQTGLVFDTSCQCCNWAARGHPFEGKPIPNPLLGLDSTRTGHHHHHH   650
CatD          --------------------PRGHPFEGKPIPNPLLGLDSTRTGHHHHHH   580
CatDΔ21       --------------------PRGHPFEGKPIPNPLLGLDSTRTGHHHHHH   559
CatDΔ46       --------------------PRGHPFEGKPIPNPLLGLDSTRTGHHHHHH   534
```

Supplementary Figure S9. Alignment of the deduced amino acid sequences of PA-full-length Chia, PA-CatD, PA-CatDΔ21 and PA-CatDΔ46. Cysteine residues are highlighted by green color.

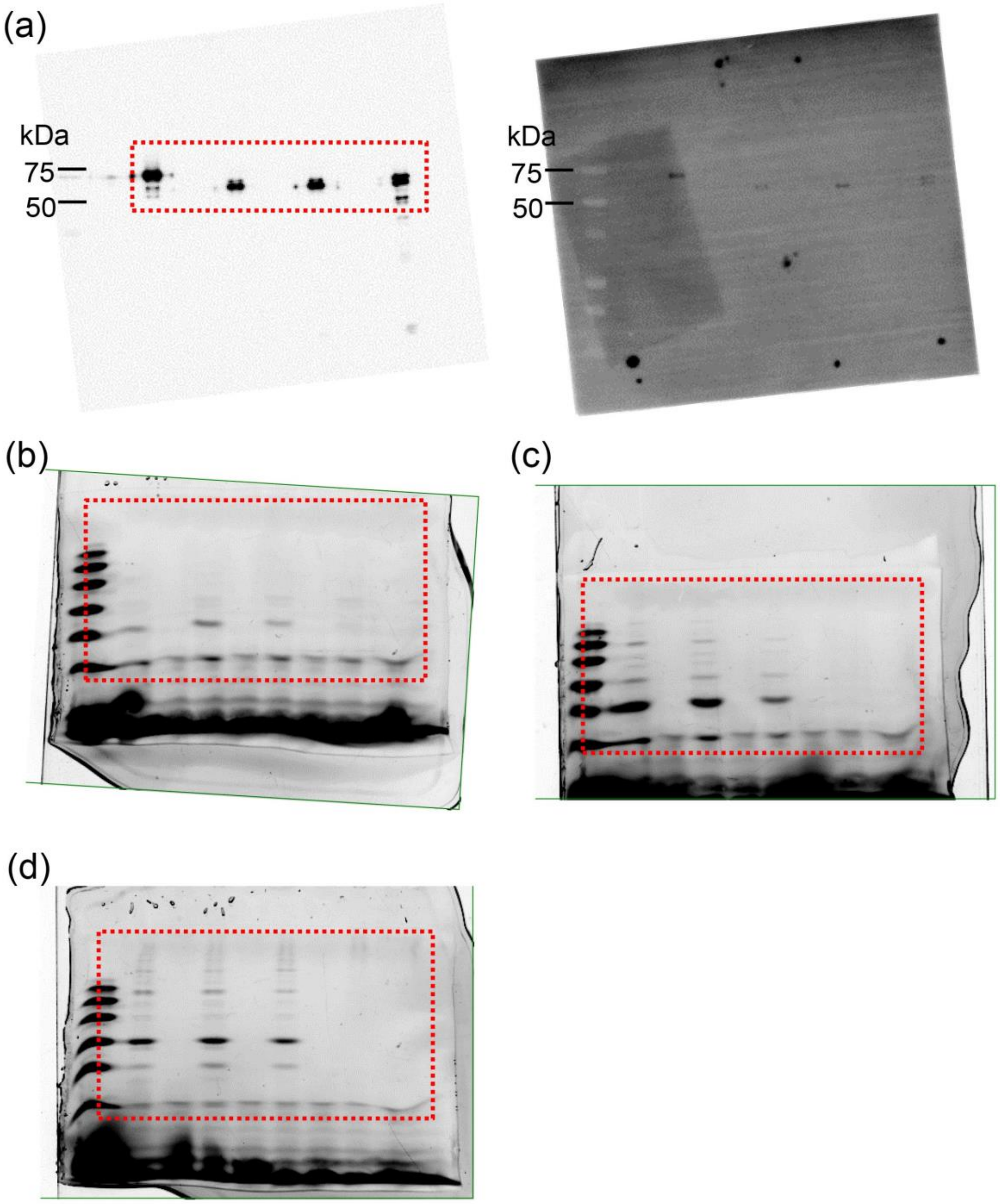

Supplementary Figure S10. Full-length gel and blots shown in Fig. 5. (a) Western blotting using anti-V5 antibody. (b-d) Full-length gel images of the FACE methods in Fig. 5b-d.

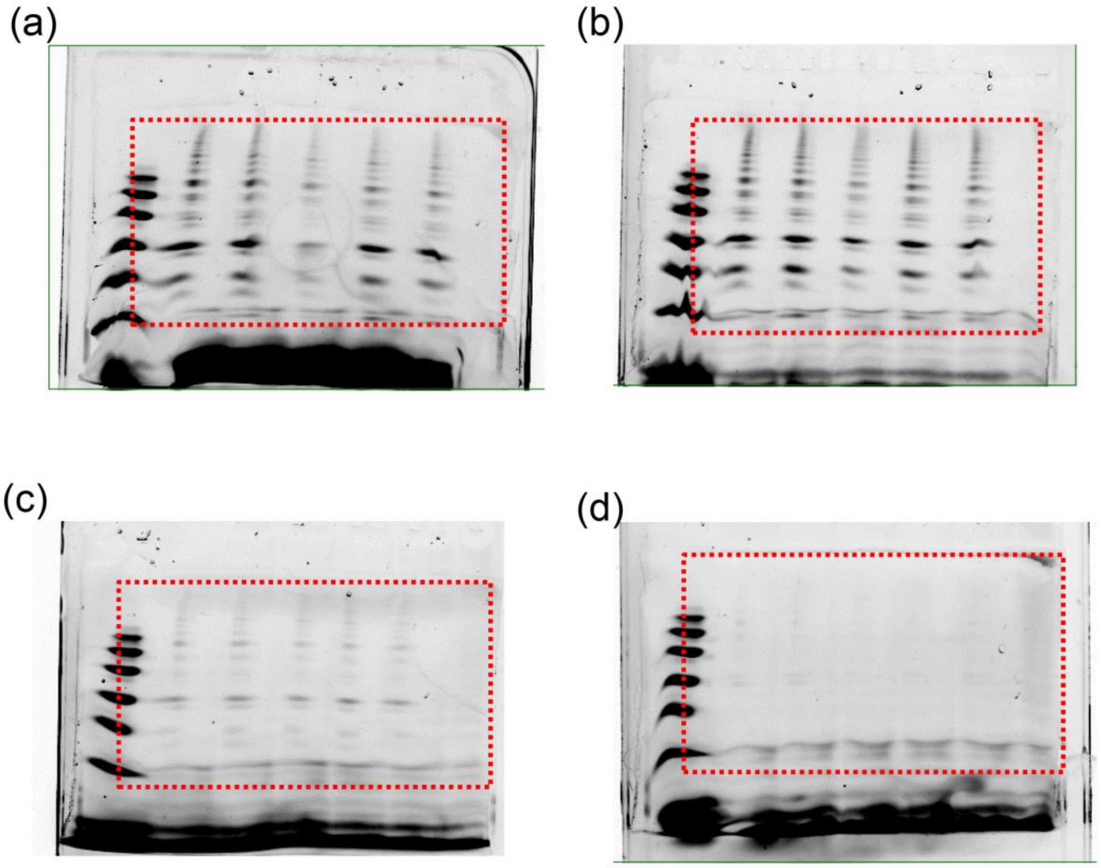

Supplementary Figure S11. Full-length gel and blots shown in Fig. 6. (a-d) Full-length gel images of the FACE methods in Fig. 6a-d.

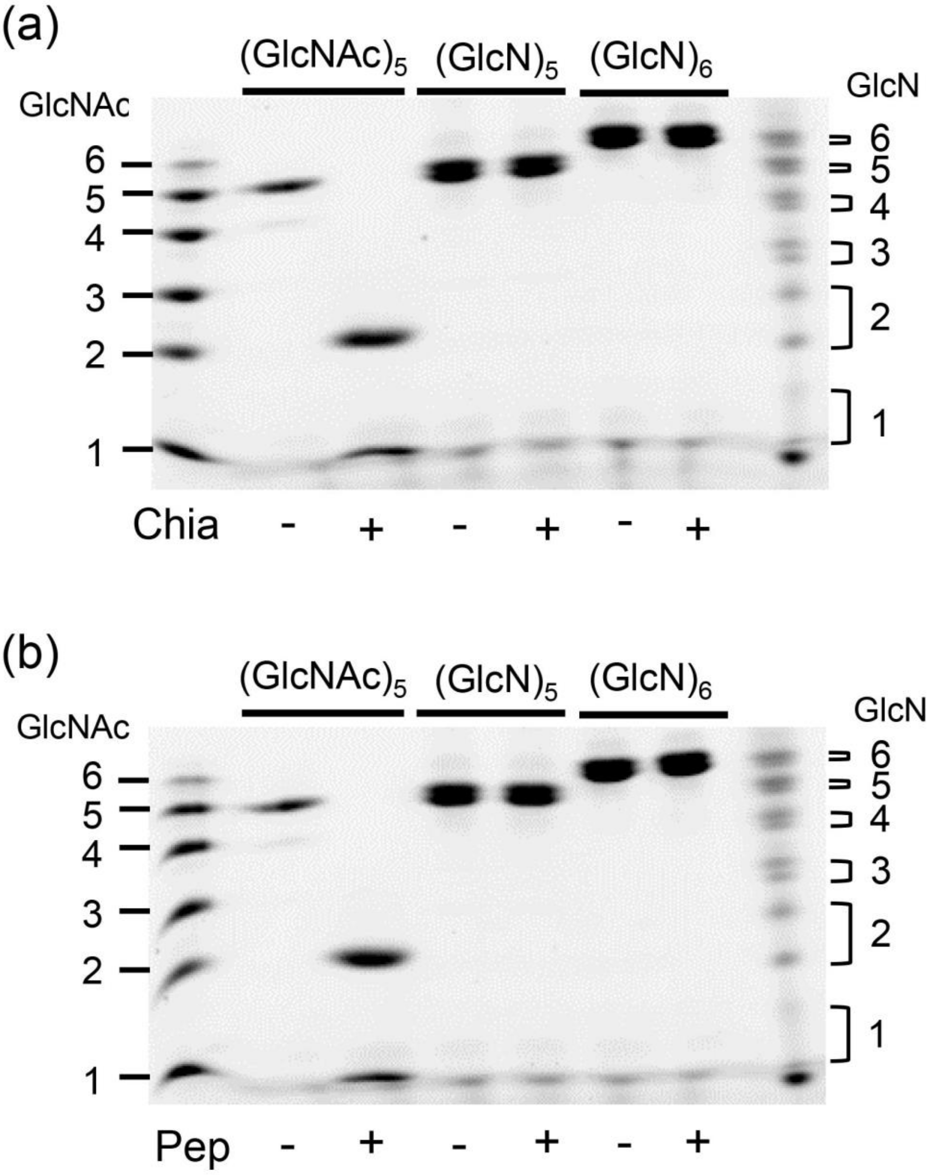

Supplementary Figure S12. Check of chitosan-degrading activity in porcine Chia and pepsin preparation using $(GlcN)_5$ and $(GlcN)_6$. $(GlcNAc)_5$, $(GlcN)_5$ or $(GlcN)_6$ were incubated with (a) porcine Chia or (b) the pepsin preparation at pH 4.0 for 16 hours. Degradation products were analyzed as described in the Methods.

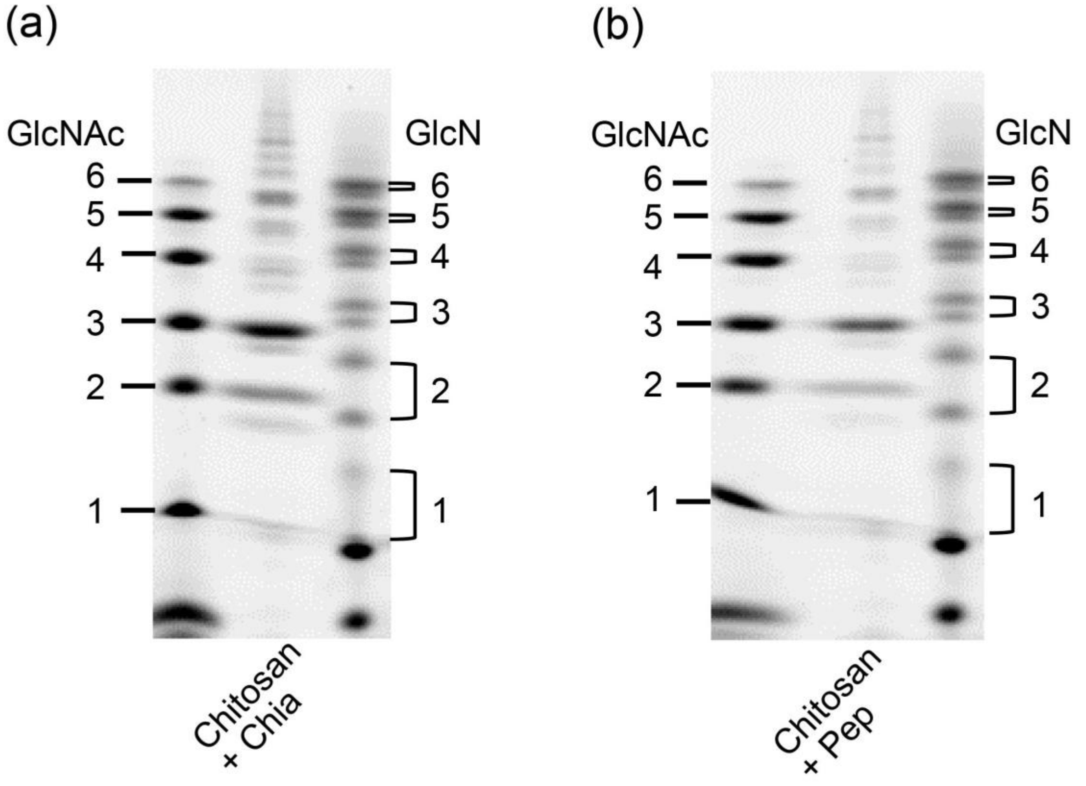

Supplementary Figure S13. Analysis of the degradation products in Fig. 6 (a). Degradation products from DD 69% chitosan were produced by incubating with (a) porcine Chia or (b) pepsin preparation at pH 4.0 for 16 hours. The products were analyzed using $(GlcNAc)_{1\text{-}6}$ (left margin) standards as well as $(GlcN)_{1\text{-}6}$ (right margin). Glucosamine (GlcN, Tokyo Chemical Industry Co., Ltd) and GlcN oligomers [$(GlcN)_{2\text{-}6}$, Seikagaku Corporation] were used as GlcN oligomer markers.

Table S1. Forward and reverse primers used to construct *E. coli*-expression vectors.

Common Forward primer
EcoRI_Pig Chia_Fw
CATGGAATTCGTACCAGCTAATATGCTACTTCACCA

For construction of pEZZ18/PA-CatD
XhoI_Pig CatD_Rv
CCTTCGAATGGGTGACCTCGAGGTCCGGTCCCGCTGCTGGGCTCACTG

For construction of pEZZ18/PA-CatDΔ21
CCTTCGAATGGGTGACCTCGAGGAAGAGCATCCTTCAGGGTGTTGATC

For construction of pEZZ18/PA- CatDΔ46
XhoI_Pig TrunCatD2_Rv
CCTTCGAATGGGTGACCTCGAGGAATGGCCCAGACCATGGCACCTCCA
